# Opportunities, challenges, and future perspectives of oncolytic virus therapy for malignant melanoma

**DOI:** 10.3389/fimmu.2025.1653683

**Published:** 2025-09-04

**Authors:** Jia-Wen Wang, Qi Feng, Jia-Hui Liu, Jian-Jun Xun

**Affiliations:** Department of Orthopedics, The Fourth Hospital of Hebei Medical University, Shijiazhuang, Hebei, China

**Keywords:** oncolytic viruses, malignant melanoma, immunogenic cell death, tumor microenvironment, innate immunity, STING pathway, dendritic cells, precision immunotherapy

## Abstract

Malignant melanoma is characterized by high heterogeneity, aggressive metastatic potential, and a profoundly immunosuppressive “cold” tumor microenvironment, contributing to broad therapeutic resistance and suboptimal responses to immunotherapy. Conventional PD-1 inhibitors yield an ORR of only 38%. As an emerging class of immunotherapeutic agents, oncolytic viruses (OV) induce ICD, promoting the release of DAMPs and activating innate immune pathways such as cGAS-STING, thereby transforming “cold” tumors into “hot” phenotypes and eliciting robust anti-tumor responses. Mechanistically, OV therapy increases the proportion of CD103^+^ dendritic cells (DCs) in lymph nodes from 5% to 25% and enhances DC–tumor synapse formation by 300%, facilitating efficient cross-presentation of tumor antigens and T-cell priming. Clinically, T-VEC combined with pembrolizumab achieves a 48.6% ORR with grade ≥3 AEs occurring in <20% of patients—superior to either monotherapy or conventional chemoradiotherapy. Nonetheless, OV therapy faces challenges including tumor heterogeneity, core mechanistic limitations, viral shedding risks, and regulatory hurdles. Over the next 5–10 years, single-cell RNA sequencing is expected to unravel molecular heterogeneity in melanoma, while CRISPR/Cas systems may enable the design of tailored OV to overcome resistance. Additional strategies such as serotype switching, JAK/STAT inhibition, and arming OV with hyaluronidase or STING agonists are under investigation to overcome immune and stromal barriers. Integration of artificial intelligence with biomarkers—such as neutralizing antibody titers, ISG expression, and STING methylation—may further enable personalized OV-based therapies. This review discusses OV therapy’s mechanisms, clinical impact, and future prospects in melanoma treatment.

## 1 Introduction

Malignant melanoma is notoriously difficult to treat due to its pronounced heterogeneity, aggressive metastatic behavior, and extensive drug resistance (1, 2). Under hypoxic microenvironmental pressures, melanoma cells can undergo phenotype switching between MITF-high and MITF-low states, contributing to a population of slow-cycling, therapy-resistant cells (1). Even with PD-1 inhibitor therapy, the objective response rate (ORR) remains limited at 38% (2), with a significant proportion of patients developing resistance, primarily attributed to the tumor’s immunologically “cold” microenvironment (3, 4). Histologically, these tumors typically exhibit low tumor mutation burden, scarce T-cell infiltration, impaired antigen presentation, and enrichment of immunosuppressive signaling molecules (4, 5)—collectively presenting major obstacles to effective immune reactivation. As a result, strategies to awaken or reprogram suppressed anti-tumor immunity have become a central focus of melanoma immunotherapy research (6, 7).

Oncolytic viruses (OV), a novel class of immunotherapeutic agents, exert effects that extend far beyond direct tumor cell lysis (8, 9). Upon infecting tumor cells, OV trigger immunogenic cell death (ICD), leading to the release of key damage-associated molecular patterns (DAMPs)—including high-mobility group box 1 (HMGB1), ATP, and calreticulin (CRT) (10, 11). These molecules activate innate immune sensors such as the STING pathway and Toll-like receptors (TLRs), initiating a robust immune cascade (10–13). Concurrently, OV remodel the tumor microenvironment, promoting M1 polarization of macrophages, enhancing natural killer (NK) cell activity, and activating dendritic cells (DCs). Notably, OV treatment elevates CD103^+^ DC levels in lymph nodes from 5% to 25% and increases the formation of DC–tumor synapses by 300%, thereby enabling efficient cross-presentation of tumor antigens and activation of cytotoxic T cells (14, 15). This sequential activation of innate and adaptive immunity positions OV as “*in situ* vaccines,” offering a promising avenue to break through the immunotherapy plateau in melanoma (8, 16).

Despite the clinical success of OV such as talimogene laherparepvec (T-VEC), which is FDA-approved for advanced melanoma (12, 17), substantial challenges remain. These include extracellular matrix (ECM) barriers limiting viral penetration, highly immunosuppressive tumor microenvironments, difficulties in accurately evaluating treatment response (e.g., >40% false positives in PET-CT), and systemic delivery inefficiencies (12, 17). These limitations must be addressed for broader and more effective application of OV therapy.

This review aims to explore four key questions:

1. Mechanistic Axis: How do OV activate innate immunity via the ICD–DAMP–STING axis? How does this pathway convert melanoma from a “cold” to “hot” phenotype, and how do tertiary lymphoid structures (TLSs) and ferroptosis contribute to sustained anti-tumor immunity?
2. Vector Comparison and Clinical Outcomes: What are the mechanistic advantages of different viral vectors (e.g., HSV-1, adenovirus, VSV)? Why does T-VEC combined with pembrolizumab achieve a 48.6% ORR, and how is its safety reflected in a <20% incidence of grade ≥3 AEs?
3. Resistance and Regulatory Challenges: How does tumor heterogeneity impact treatment response (e.g., differential progression-free survival in BRAF-mutant vs. wild-type patients)? How do neutralizing antibody clearance, type I IFN/ISG overexpression, and stromal barriers cooperatively limit OV efficacy? What are the clinical safety and regulatory concerns associated with viral shedding?
4. Future Innovations: How can single-cell RNA sequencing and CRISPR/Cas gene editing technologies address melanoma heterogeneity? How might serotype switching, JAK/STAT inhibitors, and “armed” OV (e.g., hyaluronidase, STING agonists) overcome core mechanistic limitations? How can artificial intelligence integrate biomarkers such as NAb titers, ISG expression, and STING methylation to enable personalized therapeutic strategies and predictive modeling?

**Figure 1:**
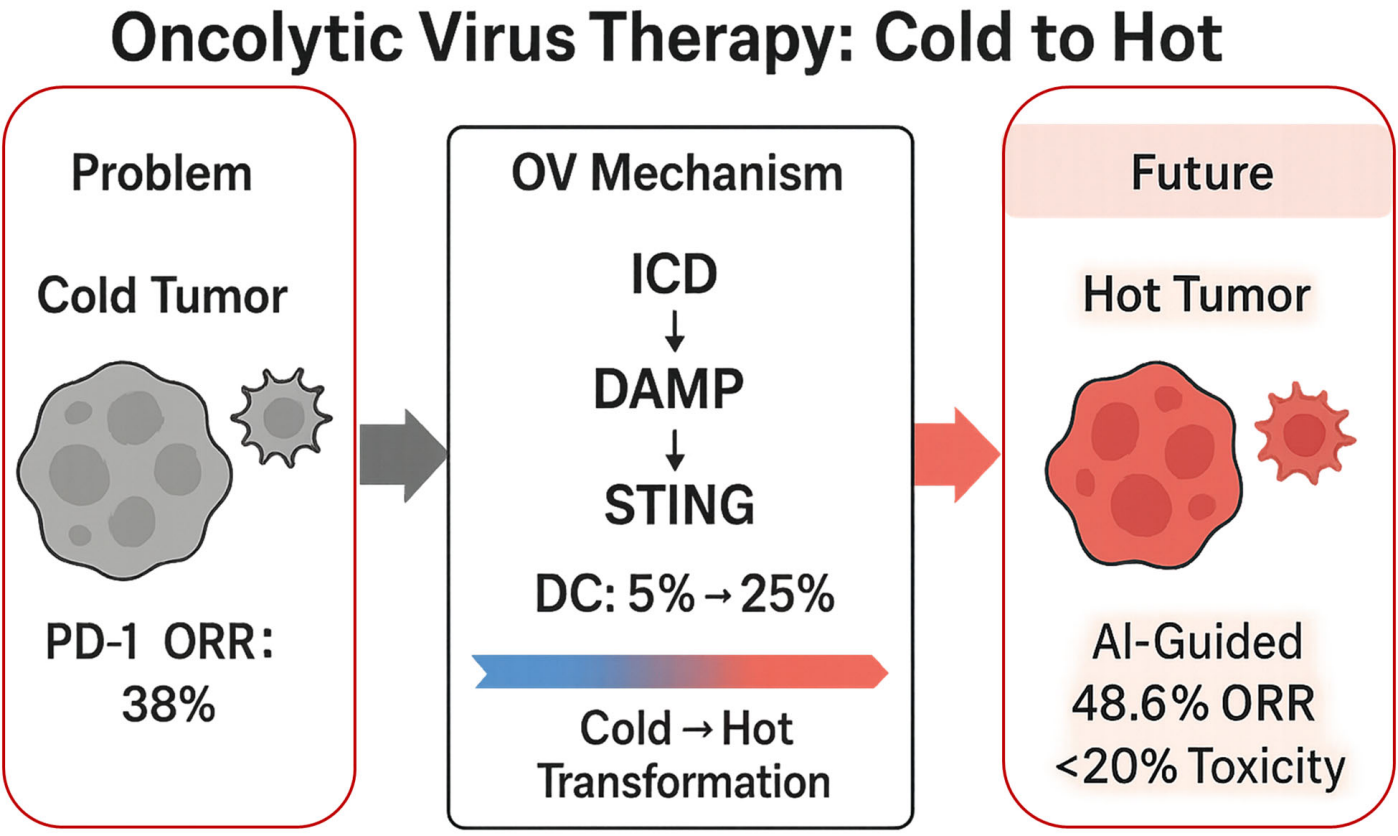
Oncolytic virus therapy transforms “cold” melanoma into “hot” tumors.

**Figure 1** provides a schematic overview of the central theme: OV-mediated reprogramming of the tumor microenvironment from “cold” to “hot.”

## 2 Oncolytic virus therapy for malignant melanoma: mechanisms and opportunities

### 2.1 Molecular immunological mechanisms of oncolytic virus therapy

Immunogenic cell death (ICD) represents a critical mechanism by which oncolytic viruses (OV) exert antitumor effects in malignant melanoma. Upon infecting tumor cells, OV induce ICD, leading to the release of key danger signals such as high mobility group box 1 (HMGB1), adenosine triphosphate (ATP), and calreticulin (CRT). These molecules act as damage-associated molecular patterns (DAMPs), which activate the host innate immune system and initiate a cascade of immune responses (18–20).

Specifically, HMGB1, normally confined to the nucleus, is released extracellularly during ICD and binds to pattern recognition receptors such as Toll-like receptors (TLRs), thereby activating downstream pathways like NF-κB and promoting proinflammatory cytokine secretion and immune cell activation (19, 20). ATP engages the P2X7 purinergic receptor, which in turn activates inflammasomes, resulting in the maturation and release of interleukin-1β (IL-1β) and recruitment of innate immune cells (19, 20). CRT translocates to the cell surface, serving as an “eat-me” signal that enhances recognition and uptake by antigen-presenting cells (APCs), particularly dendritic cells (DCs), facilitating efficient cross-presentation of tumor antigens to CD4^+^ helper T cells and CD8^+^ cytotoxic T lymphocytes (CTLs) (18).

During this process, CD4^+^ T cells secrete cytokines such as IL-2 and interferon-gamma (IFN-γ) to promote CD8^+^ T cell activation, proliferation, and differentiation, ultimately enhancing their cytolytic capacity. Upon antigen presentation, T cell receptors (TCRs) recognize the antigen–major histocompatibility complex (MHC) complex, and co-stimulatory signals such as B7-CD28 further amplify T cell activation. This results in the secretion of IFN-γ, tumor necrosis factor-alpha (TNF-α), and other cytotoxic mediators, leading to tumor cell death and the generation of long-lived central memory T cells (Tcm) and effector memory T cells (Tem), which provide durable antitumor immunity (18–20).

Moreover, the ICD process activates intracellular cytosolic DNA sensors, including the cGAS–STING pathway, which stimulates interferon regulatory factor 3 (IRF3) and NF-κB, thereby inducing type I interferons (IFNs). These cytokines boost the activity of natural killer (NK) cells and macrophages, enhancing innate immune responses and contributing to overall antitumor efficacy (19, 20).

In addition to directly killing tumor cells, OV remodel tumor immunogenicity by altering surface markers, increasing susceptibility to NK cell-mediated lysis, and recruiting macrophages. OV infection promotes the polarization of macrophages from an M2 immunosuppressive phenotype to an M1 immunostimulatory phenotype, enhancing the production of IFN-α and IFN-γ and further augmenting NK cell cytotoxicity (18, 19, 21).

Within the tumor microenvironment (TME), OV-infected tumor cells secrete immunomodulatory cytokines such as granulocyte-macrophage colony-stimulating factor (GM-CSF), IFN-α, and chemokines that facilitate immune cell recruitment while downregulating immunosuppressive cells and molecules, thereby orchestrating a comprehensive reprogramming of the TME (18, 22, 23). Specifically: GM-CSF promotes the proliferation, differentiation, and activation of granulocytes and macrophages, enhancing their phagocytic and cytotoxic functions. It also supports DC maturation, thereby improving antigen presentation and activating adaptive immune responses (18, 22, 23); IFN-α induces tumor cell apoptosis and boosts the cytotoxic functions of NK and T cells, suppressing tumor growth and proliferation (18, 22, 23); CXC chemokine ligand 10 (CXCL10) and other chemokines guide OV-activated effector T cells to the tumor site, enhancing immune infiltration and antitumor activity (18).

Additionally, OV reduce the presence and function of immunosuppressive cell types such as regulatory T cells (Tregs) and myeloid-derived suppressor cells (MDSCs), while also inhibiting the expression of programmed death-ligand 1 (PD-L1) and other immunosuppressive molecules. This release from immunosuppression restores immune cell functionality and amplifies antitumor immunity (18, 19, 22, 24, 25).

**Figure 2:**
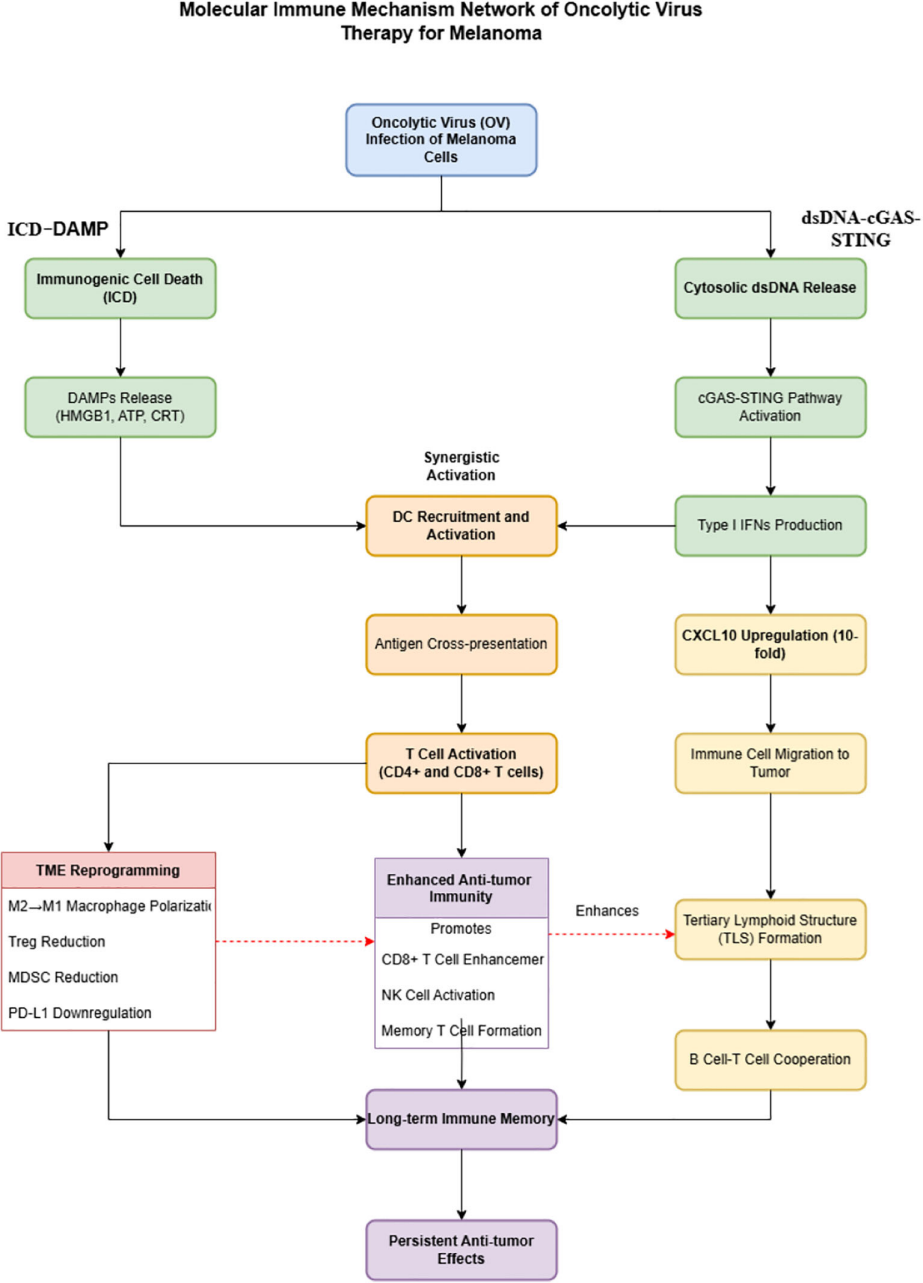
Molecular immune mechanisms network of oncolytic virus therapy for melanoma.

**Figure 2** visually summarizes this molecular network, integrating the two core mechanisms of OV therapy: ICD–DAMP axis activation and tumor microenvironment reprogramming, and elucidating their synergistic roles in initiating and sustaining robust antitumor immune cascades.

Beyond the classical ICD–DAMP axis and TME remodeling (summarized in **Table 1**), emerging molecular immune mechanisms further enhance the efficacy of OV therapy against malignant melanoma (**Table 2**): the cGAS–STING innate immune amplification mechanism is triggered by cytosolic dsDNA released during OV infection, leading to the production of chemokines such as CXCL10 and CCL5, forming an “interferon–chemokine” cascade. This process converts “cold” tumors into “hot” immunogenic phenotypes, enhancing immune infiltration and tumor clearance (26); tertiary lymphoid structure (TLS) induction remodels the tumor stroma and facilitates coordinated B/T cell responses. Notably, patients with high TLS density exhibit significantly prolonged 2-year recurrence-free survival, reaching up to 81.5% (27); ferroptosis synergy: OV efficacy is augmented by ferroptosis inducers such as Erastin, achieving tumor inhibition rates as high as 72% (28); antigen presentation and epigenetic activation: OV therapy upregulates MHC-I expression and, in combination with EZH2 inhibition, significantly enhances CD8^+^ T cell tumor recognition, thereby potentiating cytotoxic immune responses (29).

**Table 1:** Canonical mechanisms of oncolytic virus therapy in malignant melanoma.

Type	Key molecules	Mechanism	Ref.
ICD and innate immune activation	HMGB1, ATP, CRT, STING, NK cells, macrophages	Release of HMGB1, ATP, and CRT activates innate immunity; STING activation induces type I IFNs; NK cells and macrophages directly kill tumor cells	(18–20)
Tumor microenvironment (TME) modulation	GM-CSF, IFN-α, CXCL10, Tregs, MDSCs, PD-L1	GM-CSF and IFN-α activate immune cells; CXCL10 promotes T-cell infiltration; reduction of Tregs and MDSCs; PD-L1 suppression	(18, 22–24, 27)
Adaptive immunity and immunological memory	Tumor-specific antigens, CD4^+^ T cells, CD8^+^ T cells, memory T cells	Cross-presentation of tumor antigens activates T cells; activated T cells exert antitumor effects and generate long-term immune memory	(18)

Three major categories of mechanisms are covered: immunogenic cell death (ICD), TME modulation, and adaptive immune activation. HMGB1, high mobility group box 1; ATP, adenosine triphosphate; CRT, calreticulin; GM-CSF, granulocyte-macrophage colony-stimulating factor; CXCL10, CXC motif chemokine ligand 10; Tregs, regulatory T cells; MDSCs, myeloid-derived suppressor cells.

**Table 2:** Emerging molecular-immune mechanisms of OV therapy in malignant melanoma.

Mechanism type	Key factors	Mechanism	Data	Ref.
cGAS-STING innate immune amplification	cGAS, STING, TBK1, IRF3	OV-released cytosolic dsDNA activates cGAS-STING and upregulates CXCL10/CCL5, forming an “IFN-chemokine cascade” that recruits DCs and activates CD8^+^ T cells	In melanoma, STING hypermethylation leads to immune “coldness,” reversible by OV reactivation	(26)
TLS (tertiary lymphoid structure) induction	CXCL13, LTβ, CCL21	Neoadjuvant OrienX010 + toripalimab in 30 acral melanoma patients achieved 77.8% pathological response; TLS enrichment in responders indicates ECM remodeling and B/T-cell synergy	Patients with high TLS density showed 81.5% 2-year recurrence-free survival (NCT04197882)	(27)
Ferroptosis synergy	ROS, LPO, GPX4 inhibition, Erastin	Erastin-induced ferroptosis amplifies Ad5-KD01 OV efficacy, reducing tumor volume by 65% compared to monotherapy	Tumor inhibition rate in combination group: 72% (P < 0.01), with no added systemic toxicity	(28)
Antigen presentation and epigenetic activation	MHC-I/HLA-A, B, C; EZH2 inhibition	OV enhances tumor MHC-I expression and CD8^+^ T cell recognition; EZH2 inhibition relieves H3K27me3-mediated gene silencing	In vitro: HLA-ABC ↑2.3-fold, CD8^+^ cytotoxicity ↑1.9-fold	(29)

These emerging mechanisms include innate immune enhancement via cGAS-STING, TLS induction, ferroptosis synergy, and epigenetic reprogramming. TLS, tertiary lymphoid structures; ROS, reactive oxygen species; LPO, lipid peroxidation; GPX4, glutathione peroxidase 4; EZH2, enhancer of zeste homolog 2; H3K27me3, trimethylation of histone H3 at lysine 27.

### 2.2 Efficacy and safety of oncolytic virus therapy

Different oncolytic virus (OV) vectors demonstrate distinct mechanistic advantages and significant variability in efficacy and safety profiles in the treatment of malignant melanoma.

Mechanistically (**Table 3**), HSV-1–based vectors exert immune-activating effects through STING pathway activation (e.g., T-VEC) and enhanced cell-to-cell fusion (e.g., RP1). Adenoviral vectors primarily induce anti-tumor responses via cGAS–STING pathway activation and the induction of immunogenic cell death (e.g., ONCOS-102, which achieved a 70% tumor shrinkage rate), as well as through dual co-stimulatory mechanisms (e.g., LOAd703). In contrast, vesicular stomatitis virus (VSV) vectors activate innate immune signaling via the RIG-I/MDA5 axis, achieving up to a 60% tumor clearance rate (30–34).

**Table 3:** Preclinical efficacy of OV therapies in malignant melanoma.

Virus	Mechanism	Melanoma cell lines	Animal model	Evaluation methods	Key findings	Ref.
HSV-1 T-VEC	ΔICP34.5/ΔICP47 + GM-CSF; STING activation	B16F10	C57BL/6 (subcutaneous, syngeneic)	Tumor volume, OS, CD8/Treg by flow, contralateral inhibition	HSV-1 induced CXCL10, increased CD8 infiltration, inhibited contralateral tumors	(30)
HSV-1 RP1	GALV-GP-R– + GM-CSF; fusion-induced Th1 polarization	A375, SK-MEL-28	NSG-PDX + B16-F10 contralateral	Bioluminescence imaging, PD-L1 IHC, tumor volume	GALV-GP-R– enhanced immunogenicity; αPD-1 combination suppressed contralateral tumors	(31, 75)
Ad5/3 ONCOS-102	E1/E3-deleted + GM-CSF; cGAS-STING induction	A375, A2058, SK-MEL-2, SK-MEL-28	Human PBMC-human melanoma xenograft	ICD markers (CRT/ATP/HMGB1), BLI, CD8 infiltration	Tumor volume ↓70% in hu-PBMC model; ICD markers significantly increased	(32)
Ad5/35 LOAd703	CD40L + 4-1BBL; DC & CD8 activation	SK-MEL-28, B16-hCD46	SCID + B16-hCD46/C57BL/6	TIL count, ELISPOT, contralateral tumor volume	Murine LOAd703 + αPD-1/L1 led to contralateral tumor inhibition	(33, 76)
VSV-IFNβ-TYRP1	M51R mutant + hIFN-β, TYRP1; RIG-I/MDA5 driven	B16F10, B16-Ova	C57BL/6 (subcutaneous / intravenous)	Clearance rate, IFN-γ ELISPOT, ctDNA	IT+IV escalating dose achieved 60% clearance; ELISPOT↑	(34)

Various OV platforms and animal models have been employed in preclinical melanoma studies. IT, intratumoral injection; IV, intravenous injection; BLI, bioluminescence imaging; IHC, immunohistochemistry; TIL, tumor-infiltrating lymphocytes; ELISPOT, enzyme-linked immunospot; ctDNA, circulating tumor DNA; NSG, NOD/SCID/IL2Rγ; PDX, patient-derived xenograft.

Clinically (**Table 4**), the therapeutic efficacy of OV varies by vector and treatment strategy:T-VEC monotherapy has demonstrated an objective response rate (ORR) of up to 31.5% and a median overall survival (mOS) of 23.3 months. Notably, it led to a >50% reduction in lesion size in 34% of non-visceral and 15% of visceral uninjected tumors (35); RP1 combined with nivolumab in PD-1–refractory patients achieved an ORR of 32.9%, a median duration of response (mDoR) of 33.7 months, and a 1-year survival rate of 75.3%. Impressively, 96.6% of patients experienced regression in uninjected lesions, and 39.5% achieved complete lesion clearance (36); OH2 monotherapy, in patients previously treated with PD-1 inhibitors, resulted in an ORR of 58.3% and a 1-year survival rate of 94.3%, with 40% experiencing reductions in uninjected lesions (19);ONCOS-102 combined with pembrolizumab achieved a 35% ORR in PD-1–resistant patients, with 53% showing regression of uninjected lesions (37);T-VEC combined with pembrolizumab reached an ORR of 48.6%, along with a 33% reduction rate in non-injected visceral lesions (38).

**Table 4:** Clinical efficacy, systemic (abscopal) responses, and safety profile of OV therapies in melanoma.

Virus	Study type	Efficacy	Non-injected lesions / Abscopal effects	Safety indicators	Ref.
T-VEC (HSV-1 ΔICP34.5/ICP47 + GM-CSF)	Phase III OPTiM (monotherapy)	ORR 31.5%; CR 16.9%; mOS 23.3 months	≥50% reduction: non-visceral non-injected 34%; visceral 15% (e.g., liver/lung); confirmed abscopal response in 15% of patients with ≥1 distant lesion reduction	Grade ≥3 TRAEs 16.9% (mainly fever, chills)	(35)
T-VEC + Pembrolizumab	Phase III MASTERKEY-265	ORR 48.6%; CR 17.9%; PFS HR 0.86 (not significant)	Phase Ib lesion analysis: >50% reduction in injected lesions (82%), non-visceral non-injected (43%), visceral (33%); marked abscopal effect	Grade ≥3 TRAEs 20.7% (comparable to pembrolizumab alone)	(38)
RP1 (vusolimogene oderparepvec; HSV-1 + GM-CSF & GALV-GP-R)	Phase II IGNYTE (w/ Nivolumab)	ORR 32.9%; CR 15%; mDOR 33.7 months; 1-year OS 75.3%	Among RECIST 1.1 responders: ≥30% reduction in injected lesions (93.6%, 73/78) vs non-injected (79.0%, 94/119); complete clearance: 53.8% injected vs 39.5% non-injected; 96.6% showed reduction in at least one non-injected lesion	Grade ≥3 TRAEs 9.3%; no treatment-related deaths	(36)
OH2 (HSV-2 + GM-CSF)	Phase Ia/Ib (monotherapy)	ORR 37.0%; prior anti-PD-1 group ORR 58.3%; 1-year OS 94.3%; mOS 28.9 months	Phase Ib: 51 injected vs 20 non-injected lesions; shrinkage rate 51.0% vs 40.0%; maximum reduction 100%; complete disappearance of multiple distant nodules reported	No grade ≥3 treatment-related AEs observed	(19)
ONCOS-102 (Ad5/3-ΔE1 + GM-CSF)	Phase I/II pilot (w/ Pembrolizumab)	ORR 35%; mPFS/mOS still under follow-up	53% of patients showed shrinkage in ≥1 non-injected lesion, indicating systemic immune response	No dose-limiting toxicity or grade ≥3 TRAEs	(37)
V937 (Coxsackievirus A21, wild-type strain)	Phase II CALM (monotherapy)	Confirmed ORR 28.1%; 6-month PFS 38.6%; 12-month OS 75.4%	Study reported regression in non-injected lesions, but exact quantitative data not disclosed	No grade ≥3 treatment-related AEs observed	(77)

Clinical efficacy was evaluated based on various trials using RECIST/mRECIST/irRECIST criteria. mDOR, median duration of response; mOS, median overall survival; PFS, progression-free survival; HR, hazard ratio; NS, not significant; TRAE, treatment-related adverse event.

In terms of safety (**Table 4**), most OV-based regimens were well tolerated, with the incidence of grade ≥3 treatment-related adverse events (TRAEs) generally remaining below 20%. Notably, several trials reported no grade ≥3 TRAEs at all. This is in stark contrast to traditional chemotherapy and many targeted therapies, which typically report severe adverse events in 30% to 60% of cases (39–42).

## 3 Key bottlenecks and challenges in OV-based immunotherapy

In clinical practice, patient responses to oncolytic virus (OV) therapy vary significantly (23, 43, 44), primarily due to the high heterogeneity of immune status, tumor microenvironment, and tumor cell gene expression profiles (23, 43, 44). Among these, tumor heterogeneity plays a critical role in determining therapeutic outcomes (**Table 5**):Patients with BRAF wild-type tumors exhibit significantly longer disease-free survival than those with BRAF mutations (P = 0.04), with the former achieving a local objective response rate (ORR) of 80%, compared to 65% in the latter (45). Similarly, patients with high MITF expression show an ORR of 49%, whereas those with AXL-high tumors demonstrate a much lower ORR of only 15% (46). Notably, STING-low patients (~25%) can achieve 100% regression of uninjected lesions and reversal of PD-1 resistance (47), whereas in STING-high patients, the regression rate of uninjected tumors is <10% (48).

**Table 5:** Efficacy of OV therapy across distinct melanoma subtypes.

Subtype	TCGA frequency (Est.)	Study	Data	Mechanistic insight	Ref.
BRAF-V600 mutation	~38%	T-VEC ± BRAF/MEK inhibitors (NCT03088176); real-world data (n=68, BRAF-mut=31)	DFFS superior in BRAF-WT; local ORR: 80% (WT) vs 65% (mut)	MAPK hyperactivation → ↑Type I IFN, enhancing antiviral clearance; BRAF/MEKi may amplify OV replication	(45)
NRAS-Q61/K mutation	15–20%	T-VEC + MEKi (Front Oncol, n=3)	CR=1, PR=1, SD=1 (all >35 months)	MEKi enhances dsRNA/ER stress → synergistic oncolysis; brain mets remain refractory	(33)
NF1 mutation/deletion (high TMB)	12–15%	ONCOS-102 pilot: TMB-high/CD8-high → abscopal ↑	— (n=21 not stratified)	Neoantigen abundance + high CD40 → T cell recruitment; prospective validation needed	(78)
Triple-WT	15%	Real-world ORR ≈ 33%; acral/mucosal OH2 ORR 33% (n=18)	—	Heterogeneous CAR/Nectin-1, low IFN barrier → moderate HSV-2 sensitivity	(19)
MITF-high / AXL-high	MITF-high ~60%; AXL-high ~20%	ICI-treated cohort (n=84): MITF-high more responsive than AXL-high to OV/ICI	ORR: 49% (MITF-high) vs 15% (AXL-high)	MITF maintains differentiation → lower IFN resistance; AXL activates JAK/STAT → antiviral shielding	(46)
STING-low vs STING-high	~25% (STING-low/ methylated in CutMel)	STING-low: D4M3A bilateral model — T-VEC monotherapy eradicated both injected and non-injected lesions, reversing PD-1 resistance STING-high: YUMM1.7 — limited T-VEC replication; no added benefit with PD-1 blockade STING-high + JAKi: T-VEC + Ruxolitinib led to 80% tumor shrinkage at injection sites and 40% abscopal response	STING-low: 100% abscopal regression, ↑mOS by 60 days STING-high: <10% abscopal shrinkage; JAKi improved systemic effect	STING-low: delayed IFN-β → high replication + ICD STING-high: early IFN-β → ISG barrier; JAKi reopens replication window while preserving ICD	(47, 48, 68)

OV therapy responsiveness varies by melanoma subtype. TCGA, The Cancer Genome Atlas; DFFS, distant failure-free survival; ORR, objective response rate; TMB, tumor mutational burden; ICI, immune checkpoint inhibitors; CAR, coxsackie-adenovirus receptor; JAKi, JAK inhibitor; "~" denotes approximate frequency; "vs" indicates comparison.

Beyond tumor heterogeneity, fundamental mechanistic barriers also constrain OV efficacy (**Table 6**): the clearance of OV by pre-existing neutralizing antibodies (NAbs) significantly impairs therapeutic effectiveness. Patients with high baseline NAbs exhibit a median overall survival (mOS) of only 12.5 months, whereas those with low titers reach 21.2 months (HR ≈ 2.0) (49); intrinsic antiviral pathways limit viral propagation. High expression of type I interferons (IFNs) and interferon-stimulated genes (ISGs) in melanoma cells activates the IFNAR–JAK/STAT pathway, upregulating PD-1 and enhancing endogenous antiviral defenses. Consequently, ISG-high patients show a much lower response rate (15%) compared to ISG-low patients (45%) (50, 51); the tumor microenvironment (TME) also suppresses OV efficacy. Specifically, PD-L1 in the TME induces an immunosuppressive milieu via the glycolysis–lactate axis, inhibiting IFN-β production and dampening immune activation (50, 51).

**Table 6:** Key mechanistic barriers to OV therapy and corresponding strategies.

Challenge	Data	Research progress	Strategic countermeasures	Ref.
Host neutralizing antibodies (NAb) & innate viral clearance	In DIPG trials, patients with high pre-existing Ad5 NAb had shorter mOS (12.5 vs 21.2 months; HR ≈ 2.0) using Δ24-RGD OV	Δ24-RGD-H43m (serotype-switched vector) for Ad5-exposed patients under IND submission	Serotype substitution, polymer shielding, MSC carriers, sequential serological monitoring	(49)
Intracellular resistance via Type I IFN / ISG upregulation	Nat Commun 2024: IFNAR-JAK/STAT axis drives PD-1 upregulation in melanoma cells; JEM 2024: PD-L1 suppresses intracellular IFN-β via glycolysis-lactate axis; ISG-high tumors show poorest response	IGNYTE biomarker analysis: ORR ≈ 45% in ISG-low vs ≈15% in ISG-high patients	JAK/STAT inhibition, epigenetic modulation (EZH2/HDAC), or IFN antagonists (e.g., V protein, B18R) within OV genome	(50, 51)
Tumor stromal barriers limiting viral diffusion	VV-Hyaluronidase degraded HA (↓70%) and increased CD8^+^ T cell infiltration 3.2×; B16F10 model showed ≈70% tumor suppression	VV-Hyal + anti–PD-1 combo entering Phase I/II (Melanoma cohort, IND #16572)	Insertion of hyaluronidase or MMP-9 into HSV/AdV; co-delivery of ECM-degrading enzymes with OV	(52)
Immunosuppressive TME and cold phenotype	cGAS-STING hypermethylation → immune cold state; OV-induced cytosolic dsDNA can restore this axis (↑CXCL10/CCL5); oHSV-OrienX010 + Toripalimab neoadjuvant trial (NCT04197882): TLS enrichment with 77.8% pathological response	RP1 + Nivolumab (IGNYTE) in anti–PD-1 failure cohort: ORR 33.6%, CR 14.7%, median DOR >35 months, well-tolerated	Engineering of STING agonists, CD40L, IL-12 into OV; combination with LAG-3/TIGIT blockade; neoadjuvant TLS induction	(26, 65)

Summary of mechanistic barriers and corresponding therapeutic strategies. NAb, neutralizing antibody; OS, overall survival; HR, hazard ratio; ISG, interferon-stimulated gene; ORR, objective response rate; HA, hyaluronic acid; ECM, extracellular matrix; TLS, tertiary lymphoid structure; DOR, duration of response.

In addition, the stromal barrier contributes to immune exclusion. For instance, VV-Hyal—a hyaluronidase-expressing vaccinia virus—can degrade up to 70% of intratumoral hyaluronic acid (HA), resulting in a 3.2-fold increase in T cell infiltration and enhanced antitumor immunity (52). Despite these findings, the molecular mechanisms underlying these resistance factors remain incompletely understood (23, 43, 44, 53, 54).

Moreover, although OV therapies have generally demonstrated favorable safety profiles, off-target risks and viral shedding remain significant safety and regulatory concerns. For example, HSV-DNA has been detected in wound exudate dressings in up to 37% of cases, and rare instances of disseminated HSV infection have been reported even in immunocompetent patients (55, 56), indicating the potential for viral shedding and transmission. Regulatory agencies including the FDA (57), EMA (58), and PMDA (59) have acknowledged these concerns. However, >60% of OV clinical trials monitor only blood samples (60), suggesting an underestimation or neglect of shedding risk. This limited sampling approach hinders the implementation of crucial regulatory requirements, such as assessing multi-route shedding and defining sustained shedding as >3 log_10_ copies in urine/saliva/feces, which mandates isolation measures (59, 61) (**Table 7**). Therefore, the safety risks and regulatory oversight in OV therapy demand renewed scrutiny and should not be underestimated.

**Table 7:** Safety concerns (off-target effects and viral shedding) and regulatory challenges of oncolytic virus (OV) therapy, along with corresponding mitigation strategies.

Category	Evidence / guideline title	Issuing body & year	Key focus	Off-target risk	Viral shedding	Mitigation strategy	Ref.
Regulatory	Design and Analysis of Shedding Studies for Virus- or Bacteria-Based Gene-Therapy and Oncolytic Products	FDA CBER, 2019 (Current)	Specifies when and how to collect saliva, blood, urine, and injection site swabs using qPCR in preclinical and clinical phases	(1) Potential person-to-person transmission (2); Antiviral sensitivity (e.g., HSV-1 to acyclovir)	Shedding monitoring must continue until 3 consecutive negatives; protective measures for contacts required	Shedding plan and caregiver protection protocol must be submitted with the IND	(57)
Regulatory	Determining the Need for and Content of Environmental Assessments...	FDA CBER, 2015	Triggers and content requirements for Environmental Assessment (EA)	(1) Wild-type reversion or recombination (2); Animal host infection risk	Required to describe potential shedding routes and inactivation processes	EA submission mandatory if predicted shedding ≥10^8^ PFU/day	(61)
Regulatory	EMA/CAT/22473/2025: Requirements for ATMP Quality, Non-Clinical, and Clinical Data	EMA CAT/CHMP, Effective July 2025	Quality framework for ATMP clinical trials	(1) Genomic integration/germline exposure (2); Viral replication/reactivation	Section 5.3 mandates quantification of shedding curve and infectivity; long-term shedding (>90 days) must be followed	QP must include terminal inactivation validation in IMPD	(58)
Regulatory	Environmental Risk Assessment of Advanced Therapies	EMA Scientific Review, 2021	Comparative analysis of ERA practices in EU, US, and JP	Recombinant risk and aquatic viral accumulation	Products with high replication & long half-life suggested as Tier I risk; ≥2 biofluid matrices must be monitored	Offers 5-step ERA template to facilitate IND preparation	(66)
Regulatory	Specific Description of ERA for Viral Vectors	PMDA, Japan, 2024	Type-1 use approval and viral vector ERA definition	(1) Vertical transmission (germline integration) (2); Reactivation in immunosuppressed hosts	Shedding >3 log_10_ copies in urine/saliva/feces considered “persistent” and requires isolation	Emphasizes compliance with Japan’s GMO regulations for laboratory containment classification	(59)
Clinical Study	T-VEC Shedding Study in Melanoma (N=24)	Multicenter Phase I/II, 2019	HSV-1–based vector	One rare case of disseminated HSV (see B-2)	HSV-DNA detected in 37% of dressing exudates; live virus cultured in only 1.6%; urine/saliva negative	Recommend dressing change within 48 h and use of gloves by caregivers	(55)
Case Report	Disseminated HSV from T-VEC	JAAD Case Rep, 2022	Immunocompetent patient	Neuro-pseudomembranous inflammation and cutaneous spread via ICP34.5-deleted virus	Same antiviral sensitivity as wild-type HSV-1; resolved with acyclovir	FDA classified this as a reportable serious adverse event requiring annual updates	(56)
Clinical Study	ONCOS-102 Phase I (N=12)	J Immunother Cancer, 2016	Adenovirus 5/3-based vector	No observed recombination events	Urine/saliva detection 8–17%, all ≤10² genome copies; no viable virus recovered	Shedding classified as “sporadic and low risk,” yet re-evaluation recommended	(79)
Clinical Study	OH2 Phase Ia/Ib (HSV-2, N=44)	J Immunother Cancer, 2025	HSV-2–based recombinant vector	No neurotoxicity or recombination observed	Low-level viral DNA in blood/saliva; urine negative; no viable virus cultured	Recommend local injection site coverage within 24 h; BSL-2 containment advised	(19)
Preclinical	TG6002 (VACV) in Canine Safety/Shedding Model	Sci Rep, 2021	Recombinant vaccinia virus	Trace DNA in spleen, no systemic toxicity	No detectable virus in urine, saliva, or feces	Supports potential for intravenous administration	(80)
Review	Systematic Review of OV Shedding Data	Vaccines, 2023	Meta-analysis of 73 clinical trials	Summarized off-target risks: germline, recombination, environmental spread	>60% of trials only monitored blood; respiratory/fluid sampling underrepresented	Provides ERA-shedding checklist to guide sponsor regulatory planning	(60)

Summary of OV-related biosafety and regulatory challenges. FDA, Food and Drug Administration; EMA, European Medicines Agency; PMDA, Pharmaceuticals and Medical Devices Agency (Japan); CBER, Center for Biologics Evaluation and Research; EA, Environmental Assessment; ATMP, Advanced Therapy Medicinal Product; ERA, Environmental Risk Assessment; BSL-2, Biosafety Level 2; PFU, Plaque-Forming Units.

Definition of viral shedding: Detection of viral nucleic acid or live virus in body fluids.

## 4 Discussion

In the next 5–10 years, technologies such as single-cell RNA sequencing (scRNA-seq) are expected to enable a more refined understanding of gene expression profiles, cellular subtypes, and functional states of both tumor and immune cells at single-cell resolution. This will facilitate the resolution of tumor microenvironment (TME) complexity and intratumoral heterogeneity (62–64). Furthermore, molecular profiling will enable the design of OV with selective infectivity and cytotoxicity toward specific tumor cell subpopulations, thereby improving treatment precision and efficacy (62–64). Among these approaches, genome editing technologies such as CRISPR/Cas systems stand out as promising tools for enhancing the immunostimulatory potency of OV and addressing tumor heterogeneity (20, 22, 24, 63, 64).

To overcome key mechanistic barriers (**Table 6**), multiple strategies are being developed. For instance, host-mediated viral clearance due to pre-existing neutralizing antibodies (NAbs) can be mitigated by employing rare serotype recombinants such as Δ24-RGD-H43m (in Ad5-preexposed populations), polymer shielding, or mesenchymal stem cell (MSC)-based delivery platforms (49). Intracellular resistance resulting from elevated type I interferon (IFN) or interferon-stimulated gene (ISG) expression may be alleviated through combination therapies involving JAK/STAT inhibitors, EZH2/HDAC epigenetic modulators, or the insertion of IFN antagonistic genes (e.g., V protein, B18R) into the OV genome (50, 51). Dense stromal barriers may be addressed by engineering OV (e.g., HSV/AdV) to express hyaluronidase or MMP-9, co-administering ECM-degrading enzymes, or using combination therapies such as VV-Hyal with anti-PD-1 antibodies (52). To remodel immunosuppressive TMEs and convert “cold” tumors into “hot” phenotypes, arming OV with STING agonists, CD40L, IL-12, or combining them with LAG-3/TIGIT inhibitors has demonstrated therapeutic potential (26, 65).

From a safety and regulatory standpoint (**Table 7**), the next 5–10 years will see the progressive implementation of policies and clinical guidelines. Regulatory authorities such as the FDA, PMDA, and EMA are enforcing standards including the requirement for “three consecutive negative tests before sampling cessation” (57) and “isolation for patients with viral shedding >3 log_10_ copies” (59, 66). In clinical settings, protocols such as “dressing changes within 48 hours” (55) and “monitoring across ≥2 fluid matrices” (66) will help enhance biosafety awareness and improve safety evaluation frameworks. Collectively, these advances are expected to contribute to the establishment of standardized international regulations and risk-stratified safety management systems, addressing both off-target risks and viral shedding concerns associated with OV therapy.

In parallel, research into predictive biomarkers for OV therapy will continue to expand and shape clinical decision-making, especially in balancing antiviral and antitumor immune responses (**Table 8**). Predictive biomarkers such as baseline NAb titers and ISG signatures will become more widely used to anticipate treatment responses and therapeutic efficacy (67, 68). Functional biomarkers, including STING promoter methylation and MITF/AXL expression ratios, will inform virus vector selection and combination strategies (26, 69). Dynamic response biomarkers—such as post-treatment increases in CD8^+^ tumor-infiltrating lymphocytes (TILs) and PD-L1 expression, as well as the rate of circulating tumor DNA (ctDNA) clearance—will facilitate real-time efficacy assessment and dose adjustment (36). In addition, tumor mutational burden (TMB), a known double-edged biomarker, will be integrated into refined prognostic models (37).

**Table 8:** Emerging biomarkers for Oncolytic Virus (OV) therapy in malignant melanoma.

Biomarker	Study	Key findings	Underlying mechanism (Antiviral vs antitumor immunity)	Ref.
Baseline Neutralizing Antibody (NAb) Titer (≥1:64)	Pooled serological analysis + Ad5/3 OAds Phase I–II; n ≈ 120	High NAb ↑ → rapid viral clearance, ↓ ORR & OS; low/negative NAb → sustained replication and survival benefit	Prior exposure to adenovirus activates humoral antiviral barrier, hindering intratumoral reinfection	(67)
Serum IL-8 Levels and Dynamics (ELISA)	Retrospective OAd study (22 melanoma cases)	Low baseline IL-8 & post-treatment ↓ → improved mOS; post-treatment ↑ → poor prognosis	IL-8 recruits neutrophils, which suppress T cells and limit viral amplification; reduction may balance immunity	(81)
Type I IFN Gene Signature (ISG-high)	Mechanistic reviews + multi-model validation + T-VEC lesion transcriptomics	Overactivation ↓ → impairs viral replication and response; moderate activation ↑ → enhances T cell recruitment and ORR	IFN-α/β act as both antiviral barriers and antigen presentation enhancers—requiring temporal “windowed” regulation	(68)
STING Promoter Methylation / STING Expression	MSP / IHC / WES in melanoma cohorts + CRISPR models	Hypomethylation/high expression ↑ → hotter TME, more TILs and improved ORR; hypermethylation silences IFN pathway	Low STING expression dampens dsDNA-triggered antiviral and immunogenic signals; demethylating agents may restore both	(26)
MITF/AXL Phenotype	IHC / transcriptomic ratios + in vitro testing across viruses	MITF^high / AXL^low ↑ → more sensitive to HSV/OAd replication & lysis; AXL^high subtype resists VSV/T-VEC	MITF^high is linked to low IFN-I expression, allowing replication; AXL activates STAT-IFN signaling, enhancing clearance	(69)
Tumor Mutational Burden (TMB)	ONCOS-102 + anti–PD-1–resistant melanoma pilot; n=21	High TMB (median 11.3 vs 4.2 mut/Mb) ↑ → sustained CD8^+^ infiltration and 60% ORR; low TMB → 0% ORR	Elevated TMB generates neoantigens for OV-induced cross-presentation; however, excessively high TMB may trigger endogenous IFN	(37)
Post-treatment CD8^+^ TIL & PD-L1 Elevation	IHC (Baseline vs D43) in RP1 + Nivolumab (IGNYTE, n=140)	CD8^+^↑/PD-L1↑ associated with ORR 33.6%; no significant upregulation in non-responders	OV-induced lysis releases PAMP/DAMPs, activating local IFN-γ loop; PD-L1 upregulation suggests need for ICI combination	(36)
ctDNA (Tumor/Viral Sequences)	High-risk stage II–III melanoma surveillance study	Rapid ctDNA clearance ↑ → improved RFS; persistent viral DNA suggests intratumoral replication window	Dynamic ctDNA reflects both tumor burden and viral genome presence, aiding therapeutic balance monitoring	(82)

Overview of recent biomarker advances in melanoma OV therapy, focusing on the balance between antiviral and antitumor immunity.

Reading guide: “↑” indicates a biomarker level positively correlated with efficacy; “↓” indicates association with reduced efficacy.

NAb, Neutralizing Antibody; IL-8, Interleukin-8; MSP, Methylation-Specific PCR; WES, Whole-Exome Sequencing; ddPCR, Digital Droplet PCR; RFS, Recurrence-Free Survival.

The rapid advancement of artificial intelligence (AI) technologies is poised to revolutionize OV-based cancer therapy. Machine learning algorithms will integrate clinical, transcriptomic, radiologic, and biomarker datasets to generate individualized predictive models, guiding vector selection, dose optimization, and combinatorial design (62–64). When coupled with single-cell omics, AI will aid in addressing tumor heterogeneity at the cellular level (62–64). Integration of AI with CRISPR/Cas-based editing will further accelerate the development of OV with enhanced immunogenicity and safety profiles (20, 22, 24, 63, 64). Nanotechnology platforms will facilitate the co-delivery of OV with immunomodulators or chemotherapeutics, promoting synergistic therapeutic effects (62–64). The emergence of multi-targeting viral vectors will allow simultaneous infection of diverse tumor cell subsets and sustained release of immunoregulatory molecules, thereby amplifying antitumor immunity (20, 22, 24, 63, 64).

Finally, as large-scale randomized clinical trial data accumulates and AI algorithms continue to evolve, key prognostic factors—such as the optimal timing, dosage, and sequencing of OV combination regimens—will be more accurately defined (23, 53, 54, 70–74), ultimately enabling a broader population of patients with malignant melanoma to benefit from OV therapy.

Oncolytic virus (OV) therapy induces immunogenic cell death (ICD), which releases damage-associated molecular patterns (DAMPs) and activates the cGAS-STING pathway, thereby transforming immunologically “cold” tumors (with a PD-1 inhibitor objective response rate [ORR] of only 38%) into “hot” tumors. This process increases the proportion of CD103^+^ dendritic cells (DCs) from 5% to 25%. Clinically, T-VEC combined with pembrolizumab has demonstrated an ORR of 48.6% with a treatment-related serious adverse event rate below 20%. In the future, artificial intelligence (AI)-guided approaches may facilitate individualized and precise OV-based therapies.

This diagram illustrates the immunoactivation network triggered by OV therapy in melanoma. Upon infection, OV activates two parallel pathways: the left axis represents the ICD pathway, leading to the release of DAMPs, while the right axis shows the cGAS-STING pathway, upregulating type I interferons and CXCL10. Together, these pathways enhance dendritic cell activation, tumor antigen cross-presentation, and T-cell responses, and promote tumor microenvironment (TME) reprogramming and tertiary lymphoid structure (TLS) formation, establishing durable antitumor immunity. Color-coded stages include: initiation events (blue), early molecular signals (green), immune cell activation (orange), TME remodeling (red), and long-term immune memory (purple). Solid arrows indicate direct effects, and dashed arrows indicate promoting relationships.

### 4.1 Permission to reuse and copyright

Permission must be obtained for use of copyrighted material from other sources (including the web). Please note that it is compulsory to follow figure instructions.

## Data Availability

The original contributions presented in the study are included in the article/supplementary material. Further inquiries can be directed to the corresponding author.
